# Amplifying Lateral Flow Assay Signals for Rapid Detection of COVID‐19 Specific Antibodies

**DOI:** 10.1002/gch2.202200008

**Published:** 2022-05-11

**Authors:** Rowa Y. Alhabbab, Mohamed A. Alfaleh, Reem M. Alsulaiman, Sawsan S. Alamri, Mais S. Eyouni, M‐Zaki ElAssouli, Adel M. Abuzenadah, Anwar M. Hashem

**Affiliations:** ^1^ Vaccines and Immunotherapy Unit King Fahd Medical Research Center and Department of Medical Laboratory Sciences Faculty of Applied Medical Sciences King Abdulaziz University Jeddah 21589 Saudi Arabia; ^2^ Vaccines and Immunotherapy Unit King Fahd Medical Research Center and Department of Pharmaceutics Faculty of Pharmacy King Abdulaziz University Jeddah 21589 Saudi Arabia; ^3^ Vaccines and Immunotherapy Unit King Fahd Medical Research Center King Abdulaziz University Jeddah 21589 Saudi Arabia; ^4^ Department of Medical Laboratory Sciences Faculty of Applied Medical Sciences King Abdulaziz University Jeddah 21589 Saudi Arabia; ^5^ Vaccines and Immunotherapy Unit King Fahd Medical Research Center and Department of Medical Microbiology and Parasitology Faculty of Medicine King Abdulaziz University Jeddah 21589 Saudi Arabia

**Keywords:** antibodies, COVID‐19, diagnostics, infection, rapid assays, SARS‐CoV‐2, serology

## Abstract

Rapid lateral flow immune‐assays are point‐of‐care diagnostic tools that are easy to use, cheap, and do not need centralized infrastructure. Therefore, these devices are appealing for rapid detection of the humoral immune responses to infections, particularly severe acute respiratory syndrome coronavirus 2 (SARS‐CoV‐2). The novel technique introduced here uses a complex of anti‐SARS‐CoV‐2 N‐protein antibodies conjugated to gold nanoparticles that are bound to five SARS‐CoV‐2 N protein conjugated to gold nanoparticles to amplify the signals obtained from the conjugated SARS‐CoV‐2 N protein and to enhance the assay detection limit. To validate the performance of the adopted lateral flow, serum from SARS‐CoV‐2 seropositive individuals and prepandamic negative samples are tested and compared to a validated enzyme‐linked immunosorbent assay (ELISA) for the detection of SARS‐CoV‐2 N protein specific IgG and IgM antibodies. The data shows that the designed lateral flow assay has an excellent sensitivity and specificity upon detecting IgM and IgG antibodies by applying only 2 µL from the serum sample to the adopted strips. Taken together, the developed lateral flow immunoassay assay provides a rapid, specific, and highly sensitive means to detect the immune responses against SARS‐CoV‐2 with only 2 µL from the serum sample.

## Graphical abstract

Lateral flow immunoassays are point‐of‐care tests that can be done within minutes to detect antigens or antibodies. Here, the conventional method to develop these assays for the detection of antibodies to SARS‐CoV‐2 is improved. This improvement enhances the assay sensitivity, specificity, and limit of detection while using a very small amount from the samples.

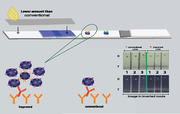

## 1 Introduction

The emergence of the severe acute respiratory syndrome coronavirus 2 (SARS‐CoV‐2) caused the devastating COVID‐19 pandemic. One of the main reason for the fast and continues spread of SARS‐CoV‐2 is the large number of the undocumented and asymptomatic individuals wherein they can spread the infection silently in the society.^[^ ^1^ ^]^ The reverse‐transcriptase (RT)‐PCR and the current rapid tests can diagnose SARS‐CoV‐2 infected individuals, however, none of these tests can provide any data on the patient immune status. Although RT‐PCR directly detects viral load, it is associated with many limitations such as the long turnaround time (≈2–10 h), high cost, and the need for highly qualified trained personal and expensive equipment. Several point‐of‐care tests (POCTs) to detect SARS‐CoV‐2 antigens such as the lateral flow immunoassays (LFIAs) have been introduced to the market to overcome some of the limitations associated with the RT‐PCR. However, their performance needs further enhancements and they do not provide any insight on patients’ immune status. Additionally, detecting the virus with RT‐PCR or by rapid testing can provide false negative results for several reasons including the timing and the quality of the collected swab samples, especially during the declining phase of the viral load in the upper respiratory tract.^[^ ^2^, ^3^ ^]^ Thus, it is critical to complement the available diagnostic assays with a rapid test that could rapidly identify new, asymptomatic and recovered COVID‐19 cases to help counteracting SARS‐CoV‐2 spread.

Individual's immune status can be detected through their IgM and IgG antibodies with any of the currently available serological tests in the market such as enzyme‐linked immunosorbent assay (ELISA) and LFIA. ELISA requires well‐trained personal in clinical laboratory settings, while LFIA can detect the presences of both IgM and IgG antibodies in a rapid and qualitative manner without the need of any specialized settings. Nonetheless, most available LFIAs require at least five microliters from the serum sample and need vigorous evaluation and validation for their sensitivity and specificity prior to their utilization to screen and identify infected or immune individuals.^[^ ^4^ ^]^ Furthermore, most LFIAs are associated with inherent low sensitivity and precision associated with the limited ability to enhance detection by enzymatic reactions for example.

SARS‐CoV‐2 infection produce antibodies against all viral antigens including both the nucleocapsid (N) and spike (S) proteins while most available vaccines for emergency use generate antibodies specific to S protein. Therefore, available SARS‐CoV‐2 LFIA devises that are specific for SARS‐CoV‐2 S protein antibodies or do not specify their targeted antigen, cannot differentiate between recently vaccinated individuals and infected or recovered patients.^[^ ^5^, ^6^, ^7^ ^]^ Thus, we developed and validated an improved LFIA method with very high sensitivity and specificity to detect SARS‐CoV‐2 N protein in serum samples with as low as 2 µL.

## 2 Results

### 2.1 Production of SARS‐CoV‐2 N Protein and Anti‐SARS‐CoV‐2 N Protein Antibodies

**Figure 1:**
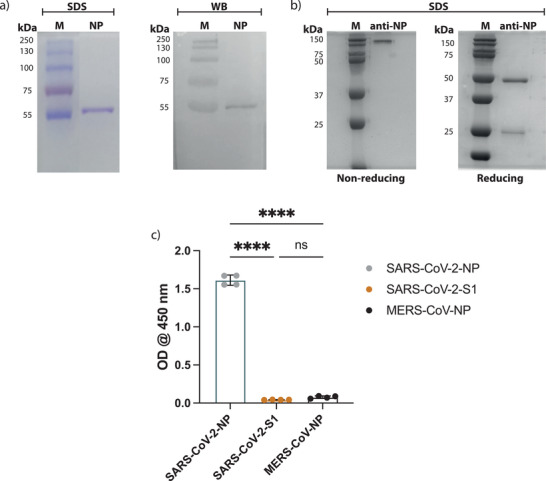
Production of SARS‐CoV‐2 N protein and anti‐SARS‐CoV‐2 N protein antibodies. a) Recombinant SARS‐CoV‐2 N protein was detected with SDS‐PAGE (on the left) and with western blot using anti‐His tag antibodies (on the right). b) SDS‐PAGE of anti‐SARS‐CoV‐2 N protein specific rabbit IgG showed the light‐chain at ≈25 kDa and the heavy chain at ≈50 kDa under reducing conditions, and the full IgG at ≈150 kDa under nonreducing conditions. c) Histogram show ELISA OD reading for the purified anti‐SARS‐CoV‐2 N protein specific antibodies upon coating the plates with SARS‐CoV‐2 N protein, SARS‐CoV‐2 S protein or MERS‐CoV N protein (*n* = 4). Statistics were calculated by unpaired *t* test and *P* values of *P < 0.05, **0.005, ***0.0005, ****<0.0005 were considered significant.

SARS‐CoV‐2 N protein is a major structural component of coronaviruses, and one of the most abundant proteins expressed upon infection, thus it is highly immunogenic.^[^ ^8^, ^9^, ^10^ ^]^ For this reason, N protein has been the target of our serological testing to detect SARS‐CoV‐2 infection. Here, we have expressed and purified a histidine‐tagged recombinant SARS‐CoV‐2 N protein in a bacterial expression system using Ni‐NTA affinity chromatography. As shown in **Figure** **1**a, the purified recombinant SARS‐CoV‐2 N protein was pure and showed the expected size of ≈46 kDa as shown by SDS‐PAGE and western blot analysis using anti‐His tag antibodies. We have previously shown that this purified protein is antigenically similar to native viral proteins as only samples from COVID‐19 seropositive but not seronegative individuals bound to the protein specifically,^[^ ^11^ ^]^ confirming that it could specifically detect SARS‐CoV‐2 antibodies in serum samples.

SARS‐CoV‐2 N protein‐specific polyclonal antibodies were obtained via precipitating rabbit antibodies that were obtained from the serum of animals using saturated ammonium sulfate solution. Then the precipitate was subsequently processed through protein A and NHS‐activated agarose coupled to SARS‐CoV‐2 N protein to isolate only N protein‐specific polyclonal IgGs. The purity of the product was then confirmed with SDS‐PAGE, which resulted in two bands under reducing conditions, (i.e., light chain band at ≈25 kDa and the heavy chain at ≈50 kDa), and one band at ≈150 kDa for the full IgG under nonreducing conditions (Figure 1b). The specificity of the antibodies was also tested with ELISA, the plates were coated with one of the following: SARS‐CoV‐2 N protein, MERS‐CoV N protein or with SARS‐CoV‐2 S1 protein, then the purified anti‐SARS‐CoV‐2 N protein antibodies were added and detected with antirabbit conjugated to HRP. The ELISA median optical density (OD) of the purified antibodies in the wells that were coated with SARS‐CoV‐2 N protein was significantly higher than the median OD reading obtained from the ELISA wells that were coated with SARS‐CoV‐2 S1 protein or MERS‐CoV N protein (Figure 1c). These results indicate that the purified antibodies were specific for SARS‐CoV‐2 N protein and do not cross‐react with the other coronavirus proteins.

### 2.2 Detection of SARS‐CoV‐2 N Protein Specific IgM and IgG Antibodies by ELISA

**Figure 2:**
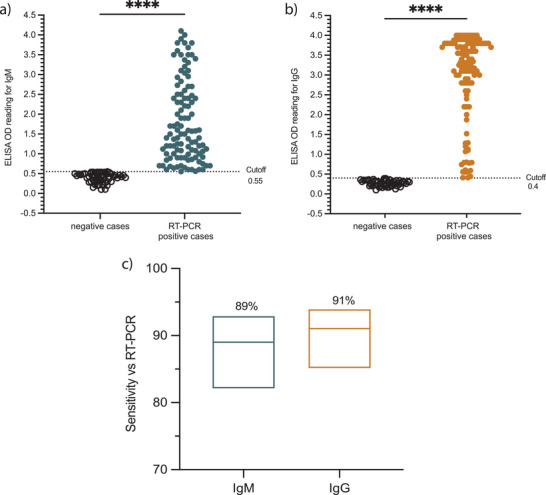
Detection of SARS‐CoV‐2 N protein specific IgM and IgG antibodies by ELISA. A total of 112 serum specimens withdrawn from RT‐PCR confirmed SARS‐Cov‐2 individuals and 52 prepandemic serum samples collected from healthy controls were tested to determine the levels of SARS‐CoV‐2‐N protein IgM and IgG antibodies with ELISA. Plots show ELISA OD reading for SARS‐CoV‐2 a) IgM and b) IgG for negative controls as well as RT‐PCR confirmed cases. The cut‐off threshold for IgM and IgG antibodies was 0.55 and 0.4, respectively. c) Floating plots show the sensitivity of the in‐house ELISA versus RT‐PCR, at the top of each plot is the sensitivity percentages and 95% confidence intervals. Statistics were calculated by unpaired t test for a and b, while Wilsom/Brown methods was used for (c), and *P* value of ^*^ *P* < 0.05, ^**^0.005, *^**^0.0005, ^****^<0.0005 were considered significant.

To characterize the antibodies profile in the samples included in this study, we determined the levels of SARS‐CoV‐2 N protein specific IgM and IgG antibodies by using in‐house ELISA that we have recently developed and validated.^[^ ^11^ ^]^ As shown in **Figure** **2**a,b, the ELISA mean OD for the used negative specimens was 0.39 (ranging from 0.1 to 0.55) for IgM and 0.29 (ranging from 0.12 to 0.4) for IgG, while the mean OD values for the positive samples that were included in this study were considerably higher for both IgM (1.79; ranging from 0.56 to 3.6) and IgG (3.06; ranging from 0.41 to 3.9). According to the precalculated cut‐off values,^[^ ^11^ ^]^ and the 112 RT‐PCR confirmed positive samples used in this study, the overall ELISA versus RT‐PCR sensitivity for the IgG was 91% (95% CI: 85–94%; 102/112) (Figure 2c). As expected, lower sensitivity value of 89% was observed for the IgM ELISA (95% CI: 82–93%; 100/112) (Figure 2c). Notably, based on our previous reported work the sensitivity of ELISA detecting antibodies was depending on the sample collection time point post symptoms onset. Moreover, these data confirm our previously observed high sensitivity of IgM/IgG ELISA based on N protein.^[^ ^4^, ^11^ ^]^

### 2.3 Development of the In‐House LFIA

**Figure 3:**
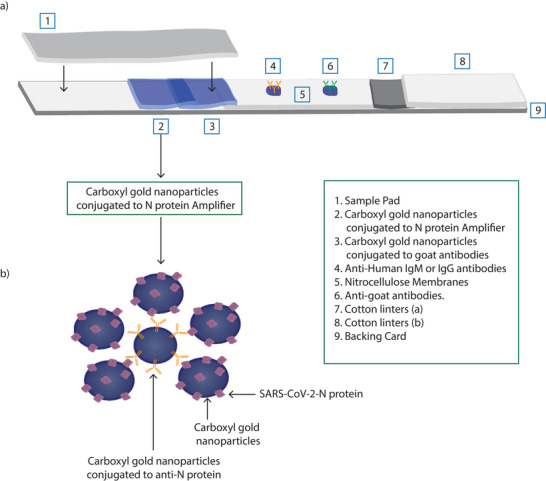
Development of the in‐house LFIA. a) Schematic of the components of the adopted LFIA strips. b) Illustration of the amplifier conjugate used in our adopted LFIA strips.

Detecting SARS‐CoV‐2‐N protein IgM and IgG antibodies in serum by ELISA, the standard method, is based on detecting the binding of these antibodies to immobilized SARS‐CoV‐2 N protein. Using the same principle, we developed LFIA to detect SARS‐CoV‐2‐N protein specific antibodies in serum that binds to SARS‐CoV‐2‐N protein conjugated to carboxyl gold nanoparticles as the mobile test phase at the test line conjugate pad and captured with either antihuman IgM or IgG antibodies on the strip test area as a solid test phase (**Figure** **3**). While several commercial antihuman IgM and IgG antibodies were tested, only one bound to the nitrocellulose membrane and gave positive signals consistently. Furthermore, we found that immobilizing either antihuman IgM or IgG antibodies but not both on the nitrocellulose membrane as a solid test phase always offered an enhanced signal. As a control, we used goat antibodies conjugated to carboxyl gold nanoparticles as the mobile control phase at the control line conjugate pad that is detected by antigoat antibodies at the strip control area as a solid control phase (Figure 3a). As such, samples could be applied so that both conjugates (test and control mobile phases) pass over the immobilized test and control areas via capillary action in the strip flow (Figure 3).

**Figure 4:**
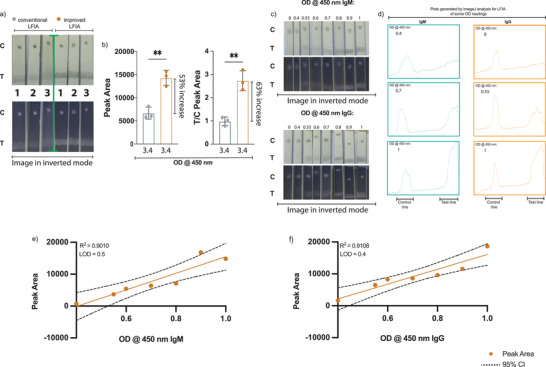
Determination of the detection limit of the in‐house LFIA. LFIA strips were developed with two method, the conventional technique that includes SARS‐CoV‐2 N protein conjugated to 150NM carboxyl gold nanoshells and our adopted method that contains premixed anti‐N protein specific antibodies conjugated to 150NM carboxyl gold nanoshells bound to SARS‐CoV‐2 N protein conjugated to 150NM carboxyl gold nanoshells at 1:5 ratio. Serum samples with known high OD reading were applied to the strips and analyzed with ImageJ software. a) Photographs of lateral flow strips show the difference in signal intensity at the test area between strips that were made with the conventional method and with the adopted improved one. b) Histogram comparing the peak area (lift) as well as T/C peak area (right) obtained from the conventional and the adopted improved LFIA strips. Serum samples of known ELISA OD reading ranging from 0 to 1, in which water was used for 0, were dispensed on the adopted LFIA strips sample loading pad. c) Photo images of lateral flow strips detecting SARS‐CoV‐2 N protein IgM (at the top) and IgG (at the bottom) antibodies tested. d) The corresponding profile plots generated with ImageJ analysis for the test area for some of the ELISA OD reading. The linear relationship between known ELISA OD reading for the e) IgM and the f) IgG antibodies and the peak area reading obtained via ImageJ analysis. Each experiment was done 3 independent times with one test per experiment. Statistics were calculated by unpaired *t* test *P* values of ^*^ *P* < 0.05, ^**^0.005, ^***^0.0005, ^****^<0.0005 were considered significant.

However, using SARS‐CoV‐2‐N protein conjugated to carboxyl gold nanoparticles resulted in a weak signal. Therefore, we tried to enhance the detection signal by increasing the amount of SARS‐CoV‐2 N protein. To this end and after several optimization steps, we used a complex consisting of purified anti‐SARS‐CoV‐2 N protein IgG antibodies conjugated to carboxyl gold nanoparticles bound to SARS‐CoV‐2 N protein conjugated to carboxyl gold nanoparticles at 1:5 ratio (1 anti‐N protein antibody conjugate:5 N protein conjugate) as the mobile test phase instead of SARS‐CoV‐2‐N protein conjugated to carboxyl gold nanoparticles (Figure 3b). Following the initial optimization experiments, 0.25 µg/5mm wide test strip and 0.5 µg mL^−1^ of immobilized antibodies including antihuman IgM, IgG antibodies as well as antigoat antibodies and mobile conjugated complex/control, respectively, were settled to detect clear signal. As a proof of concept, we compared the assay performance to strips that contains only SARS‐CoV‐2 N protein conjugated to carboxyl gold nanoparticles as a mobile phase instead of using the complex mentioned earlier in our adopted strips. Using both conjugations, 2 µL from serums with high OD reading of 3.4 upon testing with ELISA specific for SARS‐CoV‐2‐N protein antibodies were applied to each strip in triplicate followed by applying 200 µL running buffer. After 15 min, we used smartphone camera and ImageJ software to analyze the data observed, the results showed more than 2‐fold increase in the plots area values of the adopted strips compared to the conventional one (**Figure** **4**a,b). These data suggest that using the 1:5 anti‐N protein antibodies conjugate: N protein conjugate complex could amplify the detection signal by at least 2 folds or more.

### 2.4 Determination of the Detection Limit of the In‐House LFIA

Next, the limit of detections (LODs) for the improved LFIA were determined by using serum samples of known OD readings obtained from ELISA detecting SARS‐CoV‐2‐N protein IgM and IgG antibodies, applied to each strip in triplicate. As shown in Figure 4c,d, the test reliably detected SARS‐CoV‐2‐N protein IgM and IgG antibodies at ODs levels similar to the cut‐off values of the ELISA test. The LOD values as previously described is the lowest OD reading where visible band at the test area is visually detected,^[^ ^12^ ^]^ accordingly our adopted strips LOD values were seen visually at 0.55 for IgM and 0.4 for IgG. Moreover, the calibration curves shown in Figure 4e,f and Figure S1 (Supporting Information) that were obtained by plotting either the test line peak area or by plotting the T/C ratio against the ELISA OD reading shows that our LFIA for both the IgM and the IgG antibodies have a good linear dynamic. The latter ranged upon plotting test line peak area against the ELISA OD reading from 0.5 for IgM and 0.4 for IgG to 1 OD. Hence, we demonstrated a lateral flow assay that is able to detect SARS‐CoV‐2‐N protein antibodies with a better visually seen bands and with LODs identical to the standard method used to detect serum antibodies.

### 2.5 Validation of the In‐House LFIA by Testing COVID‐19 Patient Samples

**Figure 5:**
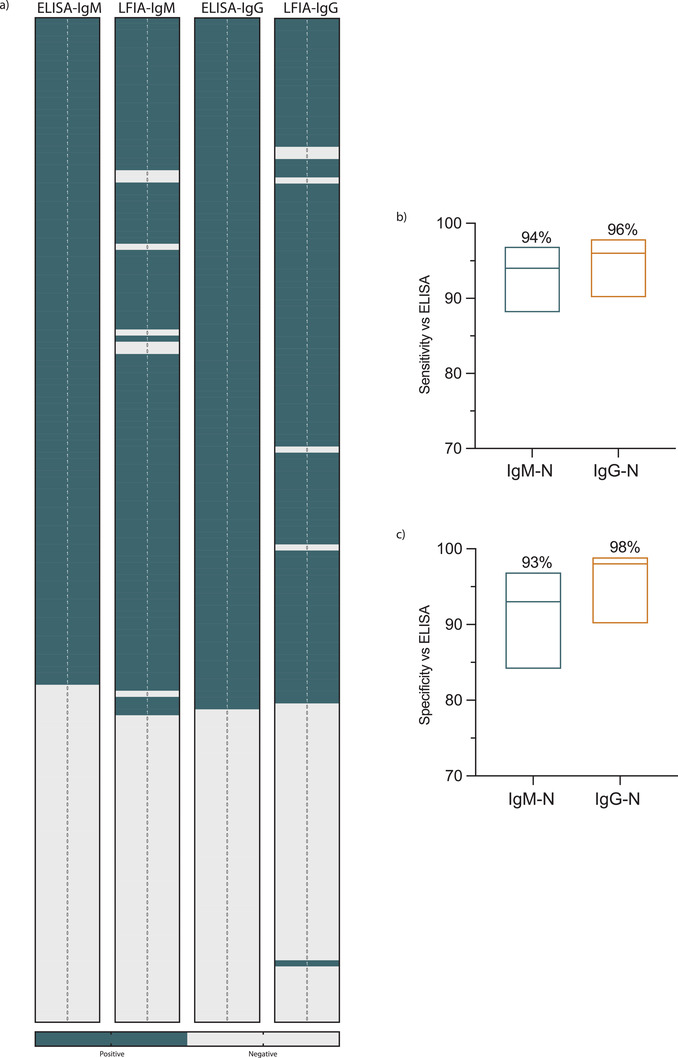
Validation of the in‐house LFIA by testing COVID‐19 patient samples. a) Heat maps representing the data that were tested by ELISA and adopted LIFA strips for both SARS‐CoV‐2 N protein IgM and IgG antibodies for 112 serums specimens of RT‐PCR confirmed to be positive for SARS‐CoV‐2 and 52 serum samples from healthy controls obtained prepandemically. b) Floating plots show the b) sensitivity and c) specificity of the adopted LFIA strips compared to ELISA, the percentages at the top of each plot and the 95% confidence intervals of IgM and IgG antibodies. Statistics were calculated by Wilsom/Brown methods.

Having established that our improved test can detect SARS‐CoV‐2‐N protein specific antibodies and based on the fact that all RT‐PCR COVID‐19 confirmed cases have developed humoral response upon testing with ELISA, we determined the assay sensitivity and specificity. Unlike ELISA results, data obtained from LFIA is qualitative, therefore, any IgM or IgG OD reading was considered positive upon exceeding the ELISA cut‐off value as a qualitative measure of antibodies. **Figure** **5**a summarizes the IgM and IgG antibodies results detected by our LFIA assays compared to ELISA. Although most of the obtained results with the improved LFIA in this study were consistent with the ELISA, several false positive as well as false negative results were observed (Figure 5a). Figure 5b shows that the sensitivity of our designed LFIA strips versus ELISA for detecting SARS‐CoV‐2‐N protein IgM antibodies was 94% (95% CI: 88–97%; 103/109) and 96% (95% CI: 90−98%; 107/112) for IgG antibodies. As for the assay specificity, it was 93% (95% CI: 84–97%; 51/55) for IgM and 98% (95% CI: 90−99%; 51/52) for IgG antibodies (Figure 5c). Thus, these data show that our adopted technique to develop improved LFIA strip is capable of producing an assay with high sensitivity and specificity compared to the standard method detecting serum antibodies, and it could generally be used to improve the performance of LFIA.

## 3 Discussion

Despite the growing number of fully vaccinated individuals, SARS‐CoV‐2 is still spreading and it appears that it will disrupt normal daily activities for years to come. As a consequence, behavioral monitoring, receiving vaccine booster shots, and regular testing are essential to counteract the virus spreading. Unfortunately, sample collection time, particularly as infection progresses, can generate false negative results by RT‐PCR, which is the most commonly used testing method, stress on the need of including serological assays to the testing strategies to improve detection sensitivity would help in minimizing the spread of infection.

Whereas all the current available vaccines produce humoral immune responses to SARS‐CoV‐2 S protein,^[^ ^5^, ^6^, ^7^ ^]^ antibodies to N protein appears only due to infection, therefore, rapid point‐of‐care serological assays for SARS‐CoV‐2 N protein specific IgM and IgG antibodies can help differentiating between vaccinated, recovered and actively infected cases. Moreover, the minimal requirements and low cost associated to preform our LFIA test would make it widely accessible to many populations, particularly those with poor‐resources. As a result, in this study we developed a rapid point‐of care test with high sensitivity of 94% and 96% for SARS‐CoV‐2‐N protein IgM and IgG antibodies, respectively, and specificity of 93% for IgM and 98% for IgG versus ELISA, standard method in measuring antibody responses. Although most of the previously published papers have shown LFIA devices detecting SARS‐CoV‐2 antibodies with various specificity and sensitivity values ranging from 69% to 100%, most of these studies had very limited numbers of samples and compared their tests with RT‐PCR confirmed cases which measures different analyte.^[^ ^13^, ^14^, ^15^, ^16^ ^]^

Up to date many variants from SARS‐CoV‐2 virus have emerged, however, most of the occurred mutations were within the viral spike protein with minimal changes in the N protein. While this might be a concern for many existing COVID‐19 vaccines and therapies, we have used in our adopted assay SARS‐CoV‐2 N protein conjugated to carboxyl gold nanoparticles to detect the humoral immune responses induced due to infection with any variants. Our data analysis from seropositive and prepandamic negative confirmed samples illustrate that our test provides clear signals at the test area with excellent sensitivity and specificity compared to ELISA. Limitation of this study is the lack of data from COVID‐19 patients that have been infected with the different variants. Although this might be valuable, as mentioned earlier the main difference between the variants exists as mutations in the spike protein, not the N protein.

Giving the importance of having LFIA to detect SARS‐CoV‐2 N protein antibodies with high sensitivity and specificity, we have developed in this study a novel technique to improve and amplify the test area signal by >50% upon comparing it to the conventional method used to produce LFIA devises. This in turn has increased test LOD to reach the cut‐off of the ELISA assay, moreover, this technology could be theoretically adopted to improve the manufacturing process of lateral flow strips to detect different analytes.

Currently, the national and international travelling situations and border control growing regulations such as vaccine status verification, quarantine requirements as well as resuming work and schools, may need regular evaluation of individual immune status to identify and differentiate immune people from infected ones. Rapid assay that can be established within 30 min maximum and requires minimum to no laboratory setting, overcome the associated challenges with the current testing system including the expensive laboratory requirement, the need of well‐trained personal and most importantly the slow turnaround time. Smartphone camera in association with a free of charge software (ImageJ) were used to confirm and record the visually obtained results are easily obtainable and do not require laboratory space, therefore, can simply be used at airports, work places and educational sites. Reporting visually generated results by LFIA and comparing the intensity of the test area to the control area have also been reported to be valid in offering sufficient information about the individual level of immunity in the absence of an electronic reading instruments.^[^ ^17^, ^18^ ^]^

## 4 Conclusion

Collectively, rapid serological assays for SARS‐CoV‐2‐N protein specific antibody testing are extremely important, therefore, we presented here a technique to develop LFIA strips that can provide strong and clear signals at the test area, have excellent sensitivity and specificity when compared to ELISA, and require very small amount of sample to be performed. Therefore, we suggest using our technique in the manufacturing process of the LFIA devises to improve the performance of the available rapid point‐of‐care serological assays.

## 5 Experimental Section

### Study Design

The LFIA prototype to detect SARS‐CoV‐2‐N protein IgM and IgG antibodies were developed under iterative steps. The capability of various versions of the adopted strips to detect SARS‐CoV‐2‐N protein IgM and IgG specific antibodies is detected by utilizing sera that were collected form 112 RT‐PCR confirmed COVID‐19 patients and from 52 healthy subjects prior to the COVID‐19 pandemic. The results obtained from the adopted LFIA prototype were compared to the data from in‐house validated ELISA to evaluate the correlation between the two assays.

### Ethics

All samples were anonymized and used based on ethical approvals obtained from the Institutional Review Board at the Ministry of Health, Saudi Arabia (IRB Numbers: H‐02‐J‐002 and Project Number: 1367), and informed consents were obtained from all participants.

### Participants

All samples were treated as infectious and hazardous, therefore, standard biosafety measures were used upon handling them. Samples were collected in yellow top collection tubes, and the serum were separated via centrifugation. The serum was then collected carefully into several Eppendorf tubes and kept at −20 °C until being used. Freezing–thawing cycles of more than 3 times were prevented. Prior to testing, samples were thaw slowly at room temperature and mixed gently.

### Recombinant SARS‐CoV‐2 N Protein Expression and Purification

Recombinant SARS‐CoV‐2‐N protein was expressed in *Escherichia coli* BL21 (DE3) cells as described previously.^[^ ^19^ ^]^ His‐tagged SARS‐CoV‐2 N protein was purified using immobilized metal affinity chromatography with a 5 mL HisTrap excel column (Cytiva, Marlborough, MA). The column was equilibrated in 20 × 10^−3^ m sodium phosphate pH 7.4 + 500 × 10^−3^ m NaCl. The harvested supernatant was loaded onto the column and then washed with equilibration buffer containing 20 × 10^−3^ m of imidazole. N protein was then eluted with 20 × 10^−3^ m of sodium phosphate pH 7.4 + 500 × 10^−3^ m NaCl + 500 × 10^−3^ m imidazole. Eluted proteins were desalted into phosphate‐buffered saline (PBS) using a HiPrep 26/10 column (Cytiva, Marlborough, MA) and then filtered using a 0.22 m filter. The positive fractions of N protein was then pooled, and after aliquoting the products, it was stored at −80 °C until used. By using SDS‐PAGE and western blot with anti‐His tag antibodies, confirmed the purity of the N protein produced.

### Rabbit Polyclonal Anti‐N Protein Antibodies Generation and Purification

For anti‐N protein specific polyclonal antibodies production, two NZW rabbits were immunized subcutaneously with purified recombinant SARS‐CoV‐2 N protein (Sino biological; China) mixed with Freund's Complete Adjuvant at 100 µg per injection and boosted twice with the same dose every two weeks in Freund's Incomplete Adjuvant. Serum was collected and ELISA was used to confirm the production of the anti‐N protein specific rabbit polyclonal antibodies. The total rabbit antibodies in the serum were precipitated with Saturated Ammonium Sulfate Solution (Thermofisher Scientific, Waltham, MA) according to the manufacturer's instructions, then to isolate rabbit IgG antibodies the precipitate was processed by using Protein A based affinity chromatography. Here, the serum was loaded into the HiTrap MabSelect SuRe column (Cytiva, Marlborough, MA), subsequently the column was washed with PBS, and the rabbit IgG antibodies were eluted using 0.1 m of glycine pH 3 and neutralized with 1 m of Tris–HCl (pH 9.0). Eluted IgG antibodies were desalted into PBS using a HiPrep 26/10 column (Cytiva, Marlborough, MA). Anti‐N protein antibodies were then purified via antigen‐immunoaffinity chromatography (NHS‐activated agarose coupled to N protein to obtain SARS‐CoV‐2‐N protein polyclonal specific antibodies) based on the manufacturer instructions (Thermofisher Scientific, Waltham, MA). Purified anti‐N IgG antibodies were then desalted into PBS and concentrated using Amicon ultra‐centrifugal filter unit (50 kDa cut‐off) (Merck Millipore) and filtered using a 0.22‐µm filter. The purity and the specificity of anti‐N protein specific polyclonal antibodies were then confirmed using SDS‐PAGE and ELISA, while the concentration of the protein was determined by Bradford assay.

### Indirect ELISA Detecting SARS‐CoV‐2‐N Protein IgM and IgG Antibodies

ELISA was performed as previously described by the group.^[^ ^11^ ^]^ The purified recombinant N protein was diluted at a concentration of 4 µg mL^–1^ in PBS and used to coat the ELISA plates overnight at 4 °C. Then, the plats were washed and blocked for 1 h at 37 °C. After washing, the serum samples were diluted at 1:100 and applied to the wells for 1 h at 37 °C. Subsequently, HRP‐conjugated antihuman IgM or IgG antibodies were added for 1 h at 37 °C. After washing the plates, 3,3′,5,5′ tetramethylbenzidine (TMB) substrate was applied and the reaction was stopped using 2 m H_2_SO_4_. Synergy 2 Multi‐Detection Microplate Reader (BioTek, Winooski, VT) was used at 450 nm to measure the absorbance.

### Production of 150NM Carboxyl Gold Nanoparticles Conjugated to N Protein Amplifier

Every five carboxyl gold nanoparticles conjugated to SARS‐CoV‐2‐N protein were mixed with one carboxyl gold nanoparticles conjugated to anti‐SARS‐CoV‐2‐N protein (1:5 ratio). The mixture was then incubated for 1 h at room temperature. After preparing the carboxyl gold nanoparticles conjugated to N protein amplifier, the mixture was used to prepare the conjugate pad.

### Production of Lateral Flow Strips

The LFIA strips consist of nitrocellulose membrane (GE Healthcare, Chicago, IL), with sample loading pad at one end, followed by mobile phase test line conjugate pad containing either SARS‐CoV‐2‐N protein conjugated to 150NM carboxyl gold nanoparticles or preincubated anti‐N protein specific antibodies conjugated to 150NM carboxyl gold nanoparticles and bound with SARS‐CoV‐2 N protein conjugated to 150NM carboxyl gold nanoparticles at 1:5 ratio, and mobile phase control line conjugate pad containing goat antibodies (abcam, UK) conjugated to 150NM carboxyl gold nanoparticles. Protein conjugation was preformed according to the manufacturer instruction (Nanocomposix, Canada). The nitrocellulose membrane contains two solid phase areas including test area that has rabbit antihuman IgM or IgG antibodies (abcam, UK), and control area that contains antigoat antibodies (abcam, UK). The strip also contains at the second end two cotton absorbent pads.

### LFIA Testing Method

Only 2 µL of the serum sample were dispensed on the sample loading pad, followed by adding 200 µL of running buffer (1× PBS, 1% Tween, 0.05 sodium azide) to the sample loading pad, so that the samples get mixed with the conjugates upon their addition and move via capillary action along the nitrocellulose membrane. If the sample contains antibodies specific for SARS‐CoV‐2‐N protein, it will bind to the conjugate and captured with the antihuman IgM or IgG antibodies striped on the test area. In the presence or absence of specific antibodies, the goat antibodies conjugate will migrate through the nitrocellulose membrane and bind to the antigoat antibodies located on the control area, and next, the remaining conjugates and sample will flow to the absorbent pad. Therefore, valid assay results required the presence of a signal at the control area, in all cases. The presence of a signal at the test as well as the control line was interpreted as positive results, while the presence of signal at the control only indicated negative results. The results were then recorded by using a smartphone (apple, USA) and the free ImageJ software.

### Statistical Analysis

The correlation between the observed OD reading for the ELISA and test line peak values analyzed by ImageJ software was evaluated by determining their linear regression and associated R2 values and presented as mean and error. Limit of detection was calculated based on the regression analysis generated by GraphPad Prism software and the following formula: LOD = 3.3 × standard deviation of the *y*‐intercept of the regression line/slop of the regression line.

The sensitivity and specificity binomial 95% CIs of the LFIA strips were determined with Wilson‐brown method and displayed as percentages with a middle line at the median. Statistical comparison between two variables were determined by using unpaired *t* test method either as mean with SD or with no error bar, while one‐way ANOVA method was used to compare between more than two variables and displayed as mean with SD. *P* value style were used to show significant differences (^*^ *P* < 0.05, ^**^0.005, ^***^0.0005, ^****^<0.0005). All the obtained numerical results were used directly for statistical analysis, and the sample size was 112 positive samples and 52 negative sample unless otherwise stated in the figure legend. Graphical presentations were generated using GraphPad Prism version 9.0.2 software (Graph‐Pad Software, Inc., CA, USA).

## Conflict of Interest

The authors declare no conflict of interest.

## Supporting information

Supporting Information

## Data Availability

The data that support the findings of this study are available from the corresponding author upon reasonable request.
