# Supplementary material for: Amplifying Lateral Flow Assay Signals for Rapid Detection of COVID‐19 Specific Antibodies
Source: Glob Chall. 2022 May 11;6(7):2200008. doi: 10.1002/gch2.202200008 (PMC9284640; doi:10.1002/gch2.202200008)
Supplement: Supplementary file 1 — Supporting Information [file GCH2-6-0-s001.pdf]

Global Challenges
Open Access

Supporting Information

for *Global Challenges,* DOI: 10.1002/gch2.202200008

Amplifying Lateral Flow Assay Signals for Rapid Detection of COVID-19 Specific Antibodies

Rowa Y. Alhabbab, Mohamed A. Alfaleh, Reem M. Alsulaiman, Sawsan S. Alamri, Mais S. Eyouni, M-Zaki ElAssouli, Adel M. Abuzenadah, and Anwar M. Hashem**

Supporting Information

Amplifying Lateral Flow Assay Signals for Rapid Detection of COVID-19 Specific Antibodies

Rowa Y. Alhabbab, Mohamed A. Alfaleh, Reem M. Alsulaiman, Sawsan S. Alamri, Mais S. Eyouni, M-Zaki ElAssouli, Adel M. Abuzenadah, and Anwar M. Hashem**

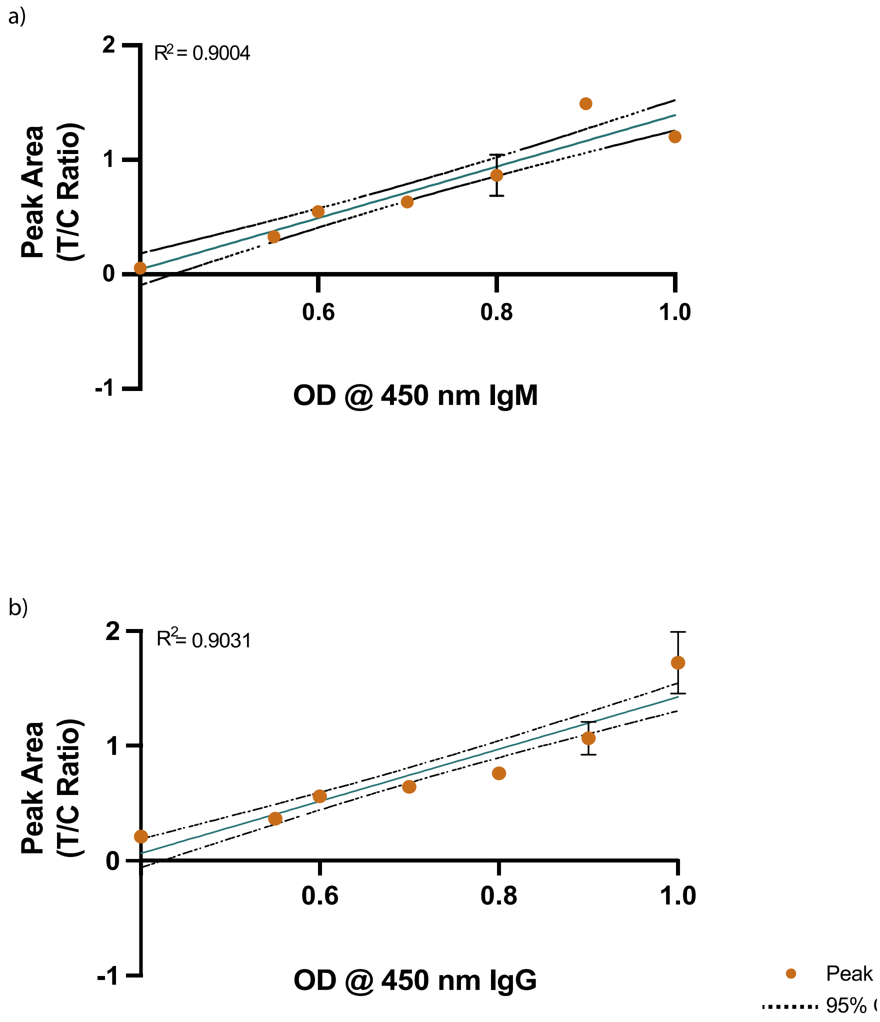

Figure S 1

Figure S1. **Determination of the detection limit of the in-house LFIA.** LFIA strips were developed with our adopted method that contains pre-mixed anti-N protein specific antibodies conjugated to 150NM carpoxyl gold nanoshells bound to SARS-CoV-2 N protein conjugated to 150NM carpoxyl gold nanoshells at 1:5 ratio. Serum samples with known high OD reading were applied to the strips and analysed with ImageJ software. (a) The linear relationship between known ELISA OD reading for the (a) IgM and the (b) IgG antibodies and the peak area reading for the T/C ratio obtained via ImageJ analysis. Each experiment was done 3 independent times with one test per experiment. Statistics were calculated by unpaired t test p values of *P<0.05, **0.005, ***0.0005, ****<0.0005 were considered significant.
